# physically interpretable residual strength prediction of corroded pipelines via symbolic Bayesian networks

**DOI:** 10.1038/s41598-026-41914-4

**Published:** 2026-03-03

**Authors:** Menghan Chen, Yuxuan Zhang, Yanchen Ye, Yuchen Lu

**Affiliations:** 1 College of Intelligent Science and Engineering, Beijing University of Agriculture, Beijing, China; 2 Yantai Research Institute, Harbin Engineering University, Yantai, China; 3 Department of Computer and Electrical Engineering, Mid Sweden University, Sundsvall, Sweden; 4 School of Mechanical-Electronic and Vehicle Engineering, Beijing University of Civil Engineering and Architecture, Beijing, China

**Keywords:** Structural health monitoring, Symbolic Bayesian networks, Corroded pipelines, Residual strength prediction, Interpretable machine learning, Engineering, Materials science, Mathematics and computing

## Abstract

Residual strength assessment of corroded pipelines is essential for ensuring the structural integrity and safe operation of gas transportation infrastructure. Traditional empirical formulas and finite element analyses, while widely used, often lack adaptability, interpretability, or computational efficiency. Recent advances in machine learning have improved prediction accuracy; however, many models remain opaque, limiting their utility in safety-critical structural health monitoring (SHM) applications where transparency and physical insight are imperative. This study introduces a novel framework, Symbolic Bayesian Networks (SyBN), for physically interpretable residual strength prediction of corroded pipelines. SyBN combines a Bayesian Feature-Weighted Neural Network (BFW-NN) for high-accuracy prediction and uncertainty quantification with a Deep Symbolic Regression (DSR) component that generates explicit mathematical expressions representing the relationship between pipeline parameters and failure pressure. A key innovation lies in an adaptive gating mechanism that dynamically balances prediction accuracy and symbolic consistency based on sample complexity. Extensive experiments were conducted on a public benchmark dataset comprising both experimental and simulation-based measurements of pipeline burst pressure. SyBN achieved state-of-the-art performance, with an $R^2$ of 0.966, RMSE of 1.304 MPa, and MAE of 0.968 MPa, outperforming several classical and ensemble learning baselines. Feature importance analysis confirmed high consistency between Bayesian-derived feature weights and SHAP values, while ablation studies validated the necessity of each framework component. The SyBN framework provides an effective and interpretable solution for residual strength prediction in corroded pipelines, offering engineers explicit symbolic models that enhance transparency and support informed decision-making. This approach aligns well with the growing demand for explainable and trustworthy machine learning in SHM tasks, particularly in critical infrastructure systems.

## Introduction

Structural health monitoring (SHM) plays a critical role in ensuring the long-term safety and reliability of infrastructure systems, particularly those operating under harsh environmental conditions^1–4^. Among them, pressurized metallic pipelines are widely used in gas and oil transportation and are often referred to as the lifeline of modern energy infrastructure^5–7^. With increasing service time, corrosion becomes one of the predominant forms of pipeline degradation, leading to wall thinning, loss of mechanical strength, and eventual failure^8,9^. Catastrophic events such as leakage and rupture can result in significant economic loss, environmental damage, and safety hazards^10–12^. Thus, accurately assessing the residual strength of corroded pipelines is essential for proactive maintenance, safety evaluation, and risk-informed decision-making in SHM.

Traditionally, residual strength assessment relies on three main categories of methods: empirical formulas, finite element analysis, and data-driven approaches^13,14^. Empirical models, often derived from fracture mechanics, offer quick estimation but are typically conservative and poorly generalizable across different geometries or material conditions^15^. FEA improves accuracy by simulating physical behavior numerically, yet it requires substantial computational effort and re-modeling when pipeline configurations or loading conditions vary^16–18^.

In recent years, machine learning has emerged as a promising tool in SHM for modeling complex, nonlinear relationships between structural parameters and damage indicators^19–23^. Algorithms such as artificial neural networks, support vector machines, random forests, and gradient boosting have shown superior predictive accuracy in estimating failure pressure or residual strength from experimental and simulated datasets^24,25^. Despite their accuracy, these models generally function as “black boxes”—they offer little insight into the physical reasoning behind their predictions^26–28^, which limits their utility in high-stakes SHM tasks where transparency and interpretability are critical.

In safety-critical scenarios such as pipeline integrity assessment, engineers require models that are not only accurate but also interpretable–providing human-readable, physically meaningful relationships between input features and structural performance metrics^29,30^. Several strategies have been developed to address this challenge. A posteriori interpretability tools, such as SHAP and LIME, can explain black-box models but do not generate self-contained physical insight^31–33^. On the other hand, intrinsically interpretable models, including decision trees and linear regression, often lack the capacity to model complex nonlinearities. Physically informed neural networks embed physics-based constraints but require predefined governing equations, which may not exist for data-driven tasks^34–36^.

Symbolic regression has thus gained attention as a method to discover closed-form mathematical expressions that fit the data while offering inherent interpretability^37,38^. By searching the function space rather than fitting predefined model forms, symbolic regression enables extraction of concise, human-understandable equations that reflect underlying physical patterns^39^. However, symbolic methods often suffer from instability and poor generalization when applied to high-dimensional, noisy data–frequent in SHM applications–and typically lag behind deep learning in prediction performance^40–42^.

To bridge the gap between interpretability and accuracy, we propose a novel Symbolic Bayesian Network (SyBN) framework tailored for residual strength prediction of corroded pipelines. SyBN integrates a Bayesian Feature-Weighted Neural Network (BFW-NN) for robust prediction and uncertainty quantification, with a Deep Symbolic Regression (DSR) module that generates explicit mathematical formulas capturing relationships among pipeline geometry, material properties, and failure pressure. An adaptive gating mechanism is introduced to dynamically balance symbolic fidelity and predictive accuracy based on sample complexity.

The key contributions of this paper are summarized as follows:

1. We construct a physically interpretable prediction framework by embedding a DSR component that simulates symbolic computation within a neural architecture, yielding explicit analytical expressions for residual strength estimation.
2. We enable synergistic optimization of accuracy and interpretability through the joint training of BFW-NN and DSR, allowing the symbolic expressions to benefit from high-performance learning while maintaining transparency.
3. We design a metacognitive gating mechanism that adaptively adjusts symbolic consistency constraints based on sample characteristics, improving model generalization and adaptability to complex data.

This work contributes to the advancement of interpretable machine learning within SHM, particularly for residual strength evaluation in corroded pipeline systems. The proposed SyBN framework supports both high-accuracy estimation and engineering interpretability, helping bridge the gap between data-driven intelligence and domain-trusted decision-making.

## Methodology

### Overall framework of SyBN for SHM applications

To address the challenge of balancing accuracy and interpretability in the residual strength assessment of corroded pipelines, this paper proposes the SyBN framework. This framework integrates two core components: a BFW-NN and DSR, achieving cooperative optimization through an adaptive gating mechanism.

Given an input vector $\textbf{x} = [D, t, \sigma _y, \sigma _u, E, d, l, w]^T \in \mathbb {R}^8$, comprising pipe geometric parameters, material properties, and defect features, SyBN predicts the residual strength of the corroded pipe, $P_b$. The BFW-NN component captures the complex nonlinear relationship between the input features and residual strengths through its Bayesian feature-weighting mechanism, while simultaneously quantifying prediction uncertainty. The DSR component constructs explicit mathematical expressions relating the input variables to the residual strengths, thereby providing a physical interpretation of the predicted results. The adaptive gating mechanism dynamically adjusts the contribution weights of the two components based on the sample features, aiming to achieve an optimal balance between prediction accuracy and interpretability. We strategically integrate BFW-NN and DSR to leverage their complementary strengths: BFW-NN effectively mitigates data noise via probabilistic weighting, while DSR–unlike traditional evolutionary algorithms–enables seamless joint gradient optimization with the predictive network. This specific configuration allows the framework to achieve high-precision nonlinear modeling while simultaneously generating interpretable physical expressions.

**Fig. 1:**
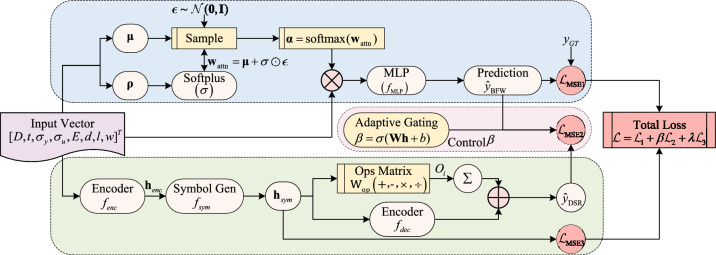
The detailed network architecture of the SyBN framework.

To clearly illustrate the internal mechanism, Fig. 1 depicts the detailed network architecture of SyBN. It demonstrates how the input vector is processed in parallel by the probabilistic BFW-NN branch and the symbolic DSR branch, with their outputs fused via the adaptive gating module to compute the total loss.

### BFW-NN: Bayesian feature-weighted neural network for robust prediction

In SHM applications, robustness and uncertainty quantification are crucial for trustworthy prediction, particularly when dealing with limited or noisy data. To this end, we design BFW-NN that models feature importance as distributions and captures predictive uncertainty. In the context of data-driven SHM, predictive uncertainty is generally categorized into aleatoric uncertainty, which arises from inherent data noise, and epistemic uncertainty, which stems from a lack of knowledge or limited training data^43^. The proposed BFW-NN primarily addresses epistemic uncertainty by treating feature attention weights not as deterministic values but as probability distributions. This allows the model to express higher confidence in regions with dense data and lower confidence in extrapolation regimes.

The distribution parameters are dynamically generated from the input features:

1 $$\begin{aligned} \boldsymbol{\mu }= & \textbf{W}_{\mu }\textbf{x} + \textbf{b}_{\mu } \end{aligned}$$

2 $$\begin{aligned} \boldsymbol{\rho }= & \textbf{W}_{\rho }\textbf{x} + \textbf{b}_{\rho } \end{aligned}$$

3 $$\begin{aligned} \boldsymbol{\sigma }= & \log (1 + \exp (\boldsymbol{\rho })) \end{aligned}$$

where $\textbf{W}_{\mu }, \textbf{W}_{\rho } \in \mathbb {R}^{8 \times 8}$ are weight matrices, $\textbf{b}_{\mu }, \textbf{b}_{\rho } \in \mathbb {R}^8$ are bias vectors, and the softplus function ensures positive variance.

During training, to allow for gradient backpropagation through stochastic nodes, attention weights are sampled using the reparameterization trick^44^:

4 $$\begin{aligned} \textbf{w}_{\text {attn}} = \boldsymbol{\mu } + \boldsymbol{\sigma } \odot \boldsymbol{\epsilon }, \quad \boldsymbol{\epsilon } \sim \mathcal {N}(\textbf{0}, \textbf{I}) \end{aligned}$$

During inference, the mean values are used for deterministic output:

5 $$\begin{aligned} \textbf{w}_{\text {attn}} = \boldsymbol{\mu } \end{aligned}$$

Attention weights are applied to the original features after softmax normalization:

6 $$\begin{aligned} \boldsymbol{\alpha } = \text {softmax}(\textbf{w}_{\text {attn}}), \quad \textbf{x}^{\prime} = \textbf{x} \odot \boldsymbol{\alpha } \end{aligned}$$

The weighted feature vector $\textbf{x}^{\prime}$ is input to the multilayer perceptron for feature extraction and prediction:

7 $$\begin{aligned} \hat{y}_{\text {BFW}} = f_{\text {MLP}}(\textbf{x}^{\prime}; \boldsymbol{\theta }_{\text {BFW}}) \end{aligned}$$

where $f_{\text {MLP}}$ is a three-layer fully connected network and $\boldsymbol{\theta }_{\text {BFW}}$ is a network parameter.

### DSR: Deep symbolic regression for interpretable modeling

Interpretable modeling is essential in safety-critical domains such as pipeline integrity assessment, where transparent reasoning supports engineering decisions. DSR module aims to generate human-readable mathematical expressions reflecting the underlying physics of failure. The DSR component employs an encoder-decoder architecture, adapted from the Deep Symbolic Regression framework^45^, to simulate the symbolic computation process within a neural network framework, thereby learning the structured mathematical relationship between input features and residual strengths.

The encoder maps the input features to the hidden space:

8 $$\begin{aligned} \textbf{h}_{\text {enc}} = f_{\text {enc}}(\textbf{x}; \boldsymbol{\theta }_{\text {enc}}) \end{aligned}$$

where $f_{\text {enc}}$ is a two-layer fully connected network. The symbol generator extracts symbol features from the encoded representation:

9 $$\begin{aligned} \textbf{h}_{\text {sym}} = f_{\text {sym}}(\textbf{h}_{\text {enc}}; \boldsymbol{\theta }_{\text {sym}}) \end{aligned}$$

DSR simulates mathematical operations through an operation weight matrix $\textbf{W}_{\text {op}} \in \mathbb {R}^{4 \times H}$, where the four rows correspond to addition, subtraction, multiplication, and division operations, and *H* denotes the symbolic feature dimension. Each operation result is computed as:

10 $$\begin{aligned} o_i = \textbf{h}_{\text {sym}}^T \textbf{w}_i, \quad i \in \{1, 2, 3, 4\} \end{aligned}$$

where $\textbf{w}_i$ represents the *i*-th row of $\textbf{W}_{\text {op}}$. The symbolic output aggregates all operation results:

11 $$\begin{aligned} y_{\text {sym}} = \sum _{i=1}^{4} o_i \end{aligned}$$

The decoder maps the symbolic features back into the target space and combines them with the symbolic output to form the final prediction:

12 $$\begin{aligned} \hat{y}_{\text {DSR}} = f_{\text {dec}}(\textbf{h}_{\text {sym}}; \boldsymbol{\theta }_{\text {dec}}) + y_{\text {sym}} \end{aligned}$$

DSR employs L1 sparsity regularization to promote concise symbolic expressions:

13 $$\begin{aligned} \mathcal {L}_{\text {sparsity}} = \lambda _s \Vert \textbf{h}_{\text {sym}}\Vert _1 \end{aligned}$$

where $\lambda _s$ denotes the sparsity penalty coefficient.

### Adaptive gating mechanism and joint loss design

SyBN achieves joint optimization through an adaptive gating mechanism. The gating unit generates dynamic constraint weights based on BFW-NN’s intermediate representation $\textbf{h}_{\text {fc3}} \in \mathbb {R}^{16}$:

14 $$\begin{aligned} {\beta } = \sigma (\textbf{W}_{\text {gate}}\textbf{h}_{\text {fc3}} + b_{\text {gate}}) \end{aligned}$$

where $\textbf{W}_{\text {gate}} \in \mathbb {R}^{1 \times 16}$ and $b_{\text {gate}} \in \mathbb {R}$ are gating parameters, and $\sigma (\cdot )$ denotes the sigmoid activation.

The joint optimization employs a multi-objective loss function:

15 $$\begin{aligned} \mathcal {L}_{\text {total}} = \mathcal {L}_{\text {MSE}}(y, \hat{y}_{\text {BFW}}) + {\beta } \cdot \mathcal {L}_{\text {MSE}}(\hat{y}_{\text {BFW}}, \hat{y}_{\text {DSR}}) + \lambda _s \Vert \textbf{h}_{\text {sym}}\Vert _1 \end{aligned}$$

This loss comprises three terms: the prediction accuracy term $\mathcal {L}_{\text {MSE}}(y, \hat{y}_{\text {BFW}})$ ensures accurate BFW-NN predictions; the consistency term ${\beta } \cdot \mathcal {L}_{\text {MSE}}(\hat{y}_{\text {BFW}}, \hat{y}_{\text {DSR}})$ aligns DSR outputs with BFW-NN; and the sparsity term $\lambda _s \Vert \textbf{h}_{\text {sym}}\Vert _1$ promotes concise symbolic expressions.

The adaptive weight ${\beta }$ dynamically adjusts symbolic consistency constraints based on sample complexity. This mechanism acts as a dynamic controller: it calculates the optimal weight $\beta$ to balance the flexibility of the neural network with the structural rigor of the symbolic regression. By doing so, it determines when to rely on data-driven fitting and when to enforce physical consistency.This self-regulating behavior embodies a metacognitive process, where the model evaluates its own prediction difficulty to adjust its strategy.

## Experimental setup and results

### Data acquisition and preprocessing

#### Data acquisition

This study utilizes a publicly available dataset^20^ sourced from existing literature for model training and validation. The dataset comprises 453 samples, encompassing 102 experimental measurements and 351 finite element analysis results. The reliability of this dataset is ensured through a rigorous validation process established in the source study. Specifically, the experimental data were obtained from full-scale burst tests conducted in compliance with industrial testing standards, while the FEA data were generated using high-fidelity numerical models explicitly calibrated against these experimental results to ensure physical consistency. Furthermore, prior to modeling, we conducted a data quality assessment to verify that all geometric and material parameters fall within physically meaningful ranges consistent with engineering practice. The input features include eight key parameters characterizing the pipe geometry, material properties, and defect characteristics: pipe diameter (*D*/mm), wall thickness (*t*/mm), yield strength ($\sigma _y$/MPa), ultimate tensile strength ($\sigma _u$/MPa), elastic modulus (*E*/MPa), defect depth (*d*/mm), defect length (*l*/mm), and defect width (*w*/mm). The target variable is the burst pressure (${P_b}$/MPa), which represents the critical pressure at which pipe damage occurs.

#### Dataset descriptive statistics and feature distributions

**Fig. 2:**
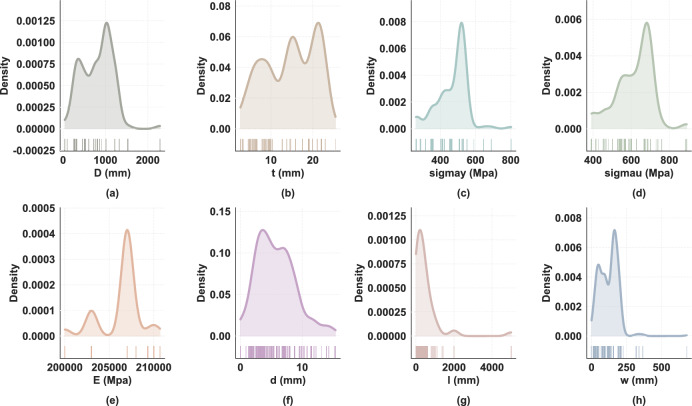
Probability density distributions of eight input variables for corroded pipeline burst pressure prediction: (**a**) pipe diameter D (mm), (**b**) wall thickness t (mm), (**c**) yield strength $\sigma$y (MPa), (**d**) ultimate tensile strength $\sigma$u (MPa), (**e**) elastic modulus E (MPa), (**f**) defect depth d (mm), (**g**) defect length l (mm), and (**h**) defect width w (mm).

The dataset employed for this study consists of 453 samples, each defined by eight input features and a single target variable, the burst pressure ($P_b$). These features characterize the pipe’s geometry (diameter *D*, wall thickness *t*), material properties (yield strength $\sigma _y$, ultimate tensile strength $\sigma _u$, elastic modulus *E*), and defect dimensions (depth *d*, length *l*, width *w*). Table 1 provides a summary of the descriptive statistics. The variables exhibit wide-ranging values, such as a pipe diameter (*D*) spanning from 41.92 mm to 2286.00 mm, reflecting diverse real-world engineering scenarios. A key challenge is presented by the target variable, burst pressure ($P_b$), which shows a high coefficient of variation (40.6%), indicating significant inter-sample heterogeneity. Further analysis of the variable distributions, as shown in Fig. 2, reveals their complex nature. For instance, the pipe diameter (*D*) follows a bimodal distribution, while several features, including material strengths and defect geometries, are characterized by pronounced right-skewness. This heterogeneity demands a modeling approach capable of handling non-Gaussian data distributions effectively.

**Table 1:** Descriptive statistics of the dataset.

Variable	Min	Max	Mean	Std
*D* (mm)	41.92	2286.00	801.88	366.38
*t* (mm)	2.73	25.40	14.55	6.01
$\sigma _y$ (MPa)	262.00	802.00	470.61	87.65
$\sigma _u$ (MPa)	392.00	891.00	616.42	96.78
*E* (MPa)	200000.00	210700.00	206340.30	2247.63
*d* (mm)	0.01	15.41	5.75	3.16
*l* (mm)	0.01	5000.00	482.69	792.15
*w* (mm)	0.01	679.80	122.46	67.56
$P_b$ (MPa)	1.37	57.33	18.17	7.38

#### Dataset correlation and interaction analysis

**Fig. 3:**
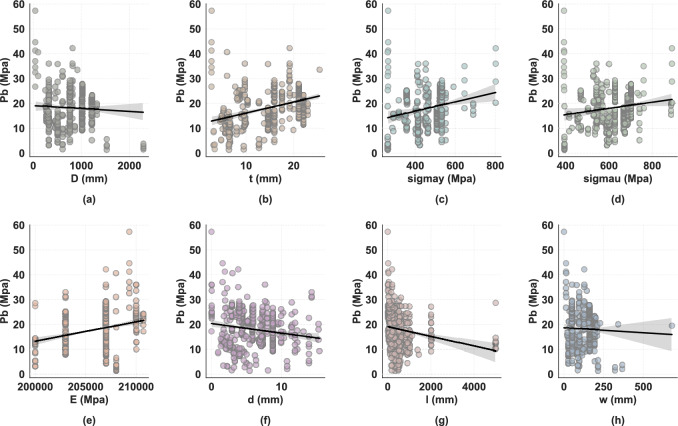
Scatter plots illustrating the relationships between input variables and burst pressure Pb for corroded pipeline strength assessment: (**a**) pipe diameter D (mm), (**b**) wall thickness t (mm), (**c**) yield strength $\sigma$y (MPa), (**d**) ultimate tensile strength $\sigma$u (MPa), (**e**) elastic modulus E (MPa), (**f**) defect depth d (mm), (**g**) defect length l (mm), and (**h**) defect width w (mm).

**Fig. 4:**
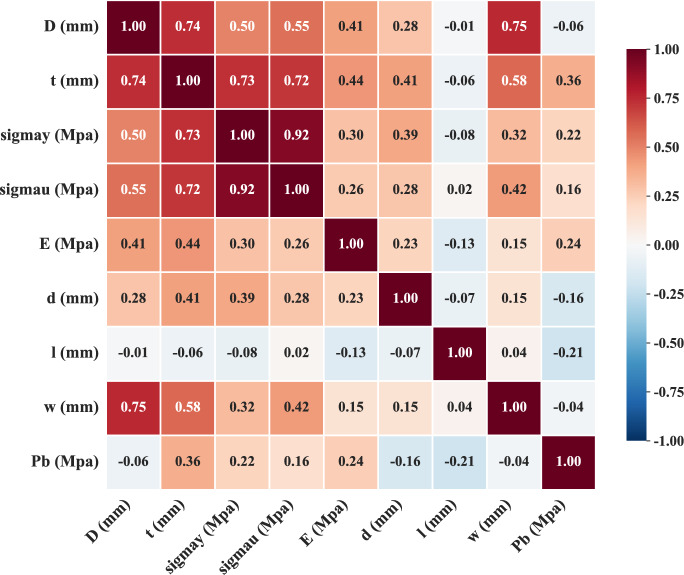
Pearson correlation heatmap quantifying the linear dependencies between input features and burst pressure.

To understand the relationships within the dataset, we analyzed both input-target correlations and inter-variable interactions. Figure 3 illustrates the complex, often nonlinear relationships between input features and the burst pressure ($P_b$). Material strength parameters ($\sigma _y$, $\sigma _u$) and defect depth (*d*) show the strongest correlations with the target, exhibiting clear positive and negative trends, respectively. However, their relationships are nonlinear, with variance increasing at higher strength levels. Other geometric parameters display weaker or non-monotonic correlations, suggesting that simple linear models would be insufficient. To quantitatively assess inter-variable dependencies, Fig. 4 presents the Pearson correlation heatmap. The matrix explicitly reveals significant multicollinearity among input features, most notably between yield strength and ultimate tensile strength ($\rho =0.92$), as well as between pipe diameter and wall thickness ($\rho =0.74$). Regarding the target variable $P_b$, wall thickness exhibits the strongest positive correlation, whereas defect length and depth show negative correlations. These statistical observations are consistent with physical failure mechanisms, where material hardening and geometric reinforcement increase load-bearing capacity, while defect expansion degrades it.

#### Data-driven modeling challenges

The comprehensive data analysis highlights three principal challenges for predictive modeling. First, the high heterogeneity of the data, evidenced by bimodal and heavily skewed variable distributions, requires a robust and flexible model architecture. Second, the complex nonlinearity in the relationships between features and the target variable necessitates a model that can capture intricate, non-monotonic trends beyond the capacity of traditional linear methods. Third, the coupled interactions among variables, characterized by an asymmetric correlation network, demand a model that can effectively learn from interdependent features without being compromised by issues like multicollinearity. These data-driven challenges collectively underscore the necessity for an advanced machine learning framework, like the proposed SyBN, which is specifically designed to address such complexities while providing interpretable insights.

### Baseline models and evaluation metrics

#### Baseline methods and comparative analysis

In order to comprehensively evaluate the performance of the proposed SyBN framework, this study compares it against a range of established machine learning methods. These baseline methods span various modeling paradigms, including linear regression techniques, instance-based algorithms, kernel-based approaches, and ensemble learning models.

- *Linear regression* Fits a linear mapping between input features and target variables using the least squares criterion.
- *Ridge regression* Enhances linear regression by incorporating an L2 regularization term, which improves model robustness and addresses multicollinearity.
- *K nearest neighbors (KNN)* A non-parametric method that makes predictions based on the characteristics of the closest training samples in feature space.
- *Support vector regression (SVR)* Utilizes kernel functions to capture nonlinear relationships within high-dimensional feature spaces, providing flexibility and precision.
- *Random forest (RF)* An ensemble learning technique that improves stability and generalization by aggregating predictions from multiple decision trees constructed on different data subsets.
- *Gradient boosted decision tree (GBDT)* Builds an additive model in a forward stage-wise fashion, where each new learner focuses on correcting the residuals of the previous ensemble.
- *Extreme gradient boosting (XGBoost)* A highly efficient implementation of GBDT that introduces regularization and parallel processing, leading to improved performance and scalability.

The key parameter configurations used in the experiments for each comparison method are summarized in Table.2.

**Table 2:** Hyperparameter settings for the baseline models experimental setup.

Method	Parameter configuration
Linear regression	No regularization
Ridge regression	L2 regularization coefficient $\alpha =0.1$ (optimized via cross-validation)
KNN	Number of neighbors $k=5$, Distance metric=Euclidean distance
SVR	Kernel function=RBF, Regularization parameter $C=1.0$, Kernel coefficient $\gamma =0.1$
RF	Number of trees=100, Maximum depth=10, Feature subset size=$\sqrt{p}$ (*p* is number of features)
GBDT	Learning rate=0.1, Number of trees=200, Maximum depth=3
XGBoost	Learning rate=0.05, Number of trees=250, Maximum depth=4, L2 regularization term $\lambda =1$

#### Evaluation metrics and statistical analysis

To ensure the robustness and statistical reliability of the results, each experiment was independently repeated ten times. The reported performance metrics represent the mean and standard deviation across these repeated runs. The evaluation of SyBN, along with all baseline methods, was conducted using three widely accepted metrics^46^:

The Coefficient of Determination ($R^2$) quantifies the proportion of variance in the target variable that is predictable from the independent variables. It is formally defined as:

16 $$\begin{aligned} R^2 = 1 - \frac{\sum _{i=1}^{n}(y_i - \hat{y}_i)^2}{\sum _{i=1}^{n}(y_i - \bar{y})^2} \end{aligned}$$

The Root Mean Square Error (RMSE) measures the standard deviation of the residuals, reflecting how concentrated the data are around the line of best fit:

17 $$\begin{aligned} \text {RMSE} = \sqrt{\frac{1}{n}\sum _{i=1}^{n}(y_i - \hat{y}_i)^2} \end{aligned}$$

The Mean Absolute Error (MAE) provides a linear score of the average magnitude of the prediction errors, offering an interpretable measure of predictive accuracy:

18 $$\begin{aligned} \text {MAE} = \frac{1}{n}\sum _{i=1}^{n}|y_i - \hat{y}_i| \end{aligned}$$

In these equations, $y_i$ denotes the true value, $\hat{y}_i$ the predicted value, and $\bar{y}$ the mean of the true values. Together, these metrics offer a comprehensive evaluation of the model’s predictive performance across multiple dimensions.

### Implementation details

Experiments were conducted on a computing platform equipped with an Intel Core i9-13980HX processor, NVIDIA GeForce RTX 4060 Laptop GPU, and 64GB memory. The PyTorch 2.0.1 deep learning framework was employed with Python version 3.9.18 and CUDA version 11.8. Key dependency libraries include numpy 1.24.3 for numerical computation and scikit-learn 1.3.0 for evaluation metric calculation. Under this configuration, the total computation time for training and evaluating the SyBN model was approximately 60 seconds. The average inference latency was negligible, satisfying the requirements for real-time SHM applications.It is worth noting that this high computational efficiency is primarily attributed to the end-to-end differentiable architecture of the proposed DSR module, which enables rapid gradient-based optimization and effectively avoids the high computational overhead associated with traditional evolutionary search strategies typically used in symbolic regression.

To strictly ensure the reproducibility of the proposed model, the detailed architectural design and training hyperparameters are summarized in Table 3. The BFW-NN consists of a three-layer multilayer perceptron with ReLU activation, while the DSR module employs a symmetric encoder-decoder structure. The sparsity regularization coefficient $\lambda _s$ was tuned to balance expression complexity and accuracy.

**Table 3:** Detailed model architecture and training hyperparameters.

Module	Parameter	Value
BFW-NN	Number of neurons (Hidden Layers)	64, 32, 16
	Activation function	ReLU
	Dropout rate	0.1
DSR	Number of neurons (Encoder/Decoder)	[8, 64, 32]/[32, 64, 8]
	Activation function	Tanh
	L1 sparsity coefficient ($\lambda _s$)	0.005
Training	Batch size	32
	Number of epochs	1000

### Comparative performance

This section evaluates the performance of SyBN on the task of corrosive pipe burst pressure prediction by comparing it with several existing machine learning methods.

Table 4 presents the average performance metrics computed from 10 independent experiments. SyBN demonstrates superior performance across all evaluated metrics: its MSE, RMSE, and MAE values are 1.700, 1.304, and 0.968, respectively, representing the lowest recorded values. Furthermore, its $R^2$ value of 0.966 is notably higher than that of other models. In contrast, integrated methods such as XGBoost and GBDT achieved higher accuracy than other baselines, but their performance was still inferior to that of SyBN. The relatively low performance of traditional linear and kernel-based methods further confirms the nonlinear characteristics inherent in this prediction task.

**Table 4:** Comparative performance of predictive models on burst pressure prediction.

Model	MSE	RMSE	MAE	$R^2$
Linear regression	25.710	5.071	3.664	0.531
Ridge	26.560	5.154	3.820	0.513
KNN	13.836	3.720	2.223	0.756
RF	9.079	3.013	1.690	0.875
SVR	30.816	5.551	3.747	0.479
GBDT	4.691	2.166	1.503	0.913
XGBoost	3.614	1.901	1.009	0.923
SyBN	1.700	1.304	0.968	0.966

**Fig. 5:**
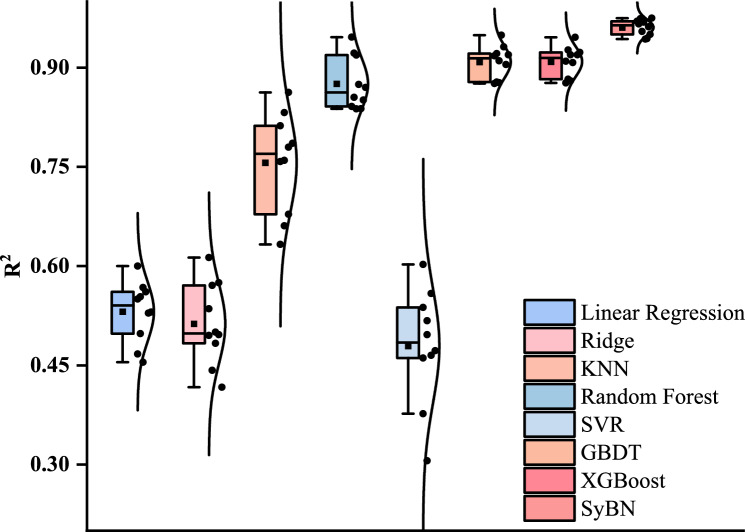
Boxplot comparison of $R^2$ performance stability across different models.

Figure 5 further elucidates the differences in model stability. As illustrated by the boxplot of the $R^2$ distribution, SyBN not only exhibits the highest median performance but also the smallest interquartile range, indicative of its excellent stability and consistency in prediction results. In contrast, the other models display greater performance fluctuations, particularly the traditional linear methods, which exhibit larger distribution dispersion, reflecting their inherent instability when addressing complex nonlinear relationships.

The high stability of SyBN originates from two protective mechanisms. The Bayesian component reduces variance by smoothing out random data noise through probabilistic weighting. Simultaneously, the symbolic component restricts the model to learn only simple and smooth mathematical forms. This prevents the network from memorizing random fluctuations and ensures consistent performance across different data samples.

### Uncertainty quantification analysis

A key advantage of the SyBN framework over traditional black-box models is its ability to quantify predictive uncertainty, encompassing both aleatoric and epistemic components. To validate this, we constructed 95% prediction intervals via 300 Monte Carlo stochastic forward passes.

**Fig. 6:**
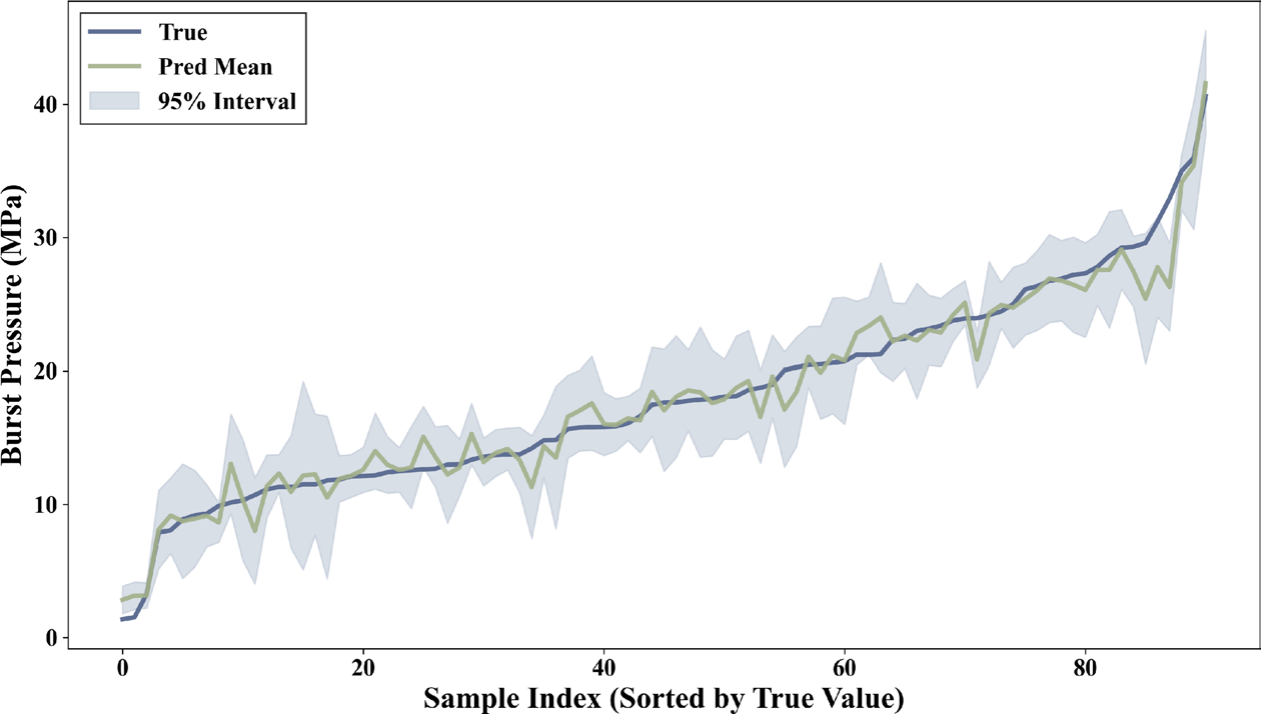
Uncertainty quantification on the test set where samples are sorted by true burst pressure. The blue line represents the true values, and the shaded area indicates the calibrated 95% prediction interval.

Figure 6 visualizes the calibrated results sorted by burst pressure. The shaded regions represent the total predictive uncertainty. It is observed that the confidence intervals effectively bracket the true values for the majority of samples. Crucially, the intervals exhibit significant widening in regions with sparse training data, correctly reflecting high epistemic uncertainty where the model lacks knowledge. Conversely, in dense data regions, the intervals narrow but maintain a baseline width, capturing the irreducible aleatoric uncertainty inherent in the stochastic nature of pipeline corrosion and material properties. This distinction allows engineers to identify whether prediction risks stem from data quality or data scarcity.

To quantitatively assess the reliability of these estimates, we calculated the Prediction Interval Coverage Probability (PICP). For a nominal 95% confidence level, the SyBN model achieved an observed PICP of 94.5%, indicating that the quantified uncertainty is well-calibrated and trustworthy for risk-informed decision-making.

### Gating mechanism analysis

To understand the decision-making of the adaptive gating unit, we analyzed the gating weight $\beta$ distribution and its relationship with prediction error.

**Fig. 7:**
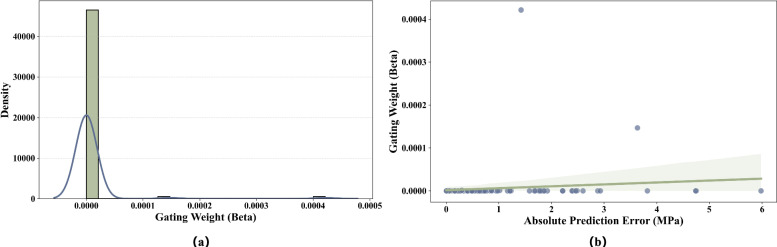
Analysis of the adaptive gating mechanism. (**a**) Density distribution of gating weight $\beta$. (**b**) Relationship between gating weight and absolute prediction error.

Figure 7 reveals two key insights into the model’s behavior. First, the overall magnitude of $\beta$ remains extremely low and stays below $5 \times 10^{-4}$. This confirms that the SyBN framework relies primarily on the high-precision BFW-NN for prediction, while the symbolic component serves as a soft auxiliary constraint rather than a dominant factor.

Second, despite these small values, Fig. 7b shows a clear positive correlation between prediction error and $\beta$. This indicates a corrective mechanism where the model automatically increases $\beta$ to enforce stricter symbolic constraints when the neural network produces large errors on difficult samples. Conversely, for easy samples where the network predicts accurately, $\beta$ drops to near zero to allow the network to fit the data flexibly without unnecessary interference.

### Ablation study

To validate the architectural design of the SyBN framework and to quantify the contribution of its principal components, a systematic ablation study was conducted. This study methodically deconstructs the model to isolate the functional significance of each module. The investigation began with the complete SyBN model (SyBN-Full), which integrates the BFW-NN, DSR, and the Adaptive Gating mechanism.

Subsequently, a series of variant models were configured to probe the necessity of each component. To ascertain the impact of symbolic regression, a variant termed SyBN w/o DSR was established by removing the DSR module and its associated symbolic consistency constraints. The role of Bayesian feature weighting was examined in the SyBN w/o BFW model, where this mechanism was substituted with a standard attention layer. To evaluate the adaptive gating, the SyBN w/o Gate model was formulated with the gating weight $\alpha$ fixed at a constant of 0.5, thereby disabling its dynamic adjustment. The influence of model parsimony on symbolic representation was assessed through the SyBN w/o Sparse configuration, from which the $L_1$ sparsity regularization in the DSR component was omitted. Finally, a Standard Neural Network (Standard NN), possessing an identical network architecture but devoid of all specialized SyBN components, served as a performance baseline.

The empirical results of the ablation experiments are presented in Table 5. The complete SyBN-Full model achieved the highest performance with a coefficient of determination ($R^2$) of 0.966. The removal of the DSR module (SyBN w/o DSR) led to a significant performance degradation of 11.6 % ($R^2=0.854$), underscoring the critical role of DSR in not only enhancing model interpretability but also imposing a structured learning paradigm that improves predictive accuracy. A comparable degradation of 12.2% was observed upon removing the Bayesian feature weighting (SyBN w/o BFW), with the $R^2$ value dropping to 0.848. This result highlights the necessity of the BFW-NN’s adaptive feature weighting for modeling complex nonlinear relationships effectively. Disabling the adaptive gating mechanism (SyBN w/o Gate) resulted in a 5.1% decrease in performance ($R^2=0.917$), confirming the importance of dynamically balancing predictive accuracy with symbolic fidelity based on sample-specific characteristics. Furthermore, the exclusion of the sparsity constraint (SyBN w/o Sparse) caused an 8.8% reduction in $R^2$ to 0.881, indicating that $L_1$ regularization is vital for learning concise and generalizable symbolic expressions. In comparison to the SyBN-Full model, the Standard NN baseline exhibited a 15.9% lower performance, which quantitatively demonstrates the synergistic advantage conferred by the integration of Bayesian modeling, deep symbolic regression, and adaptive mechanisms within the SyBN framework.

**Table 5:** Comparative analysis of ablation experiment results.

	$R^2$	Performance degradation rate (%)
SyBN-Full	0.966	–
SyBN w/o DSR	0.854	11.6
SyBN w/o BFW	0.848	12.2
SyBN w/o Gate	0.917	5.1
SyBN w/o Sparse	0.881	8.8
Standard NN	0.812	15.9

### Hyperparameter sensitivity

This section investigates the sensitivity of the SyBN model to critical hyperparameters, including learning rates and optimizers, to identify the optimal training configuration. The experiments evaluated three learning rates (0.001, 0.005, and 0.01) in combination with six optimizers (Adagrad, RMSprop, Adam, SGD, Adamax, and NAdam), resulting in 18 unique configurations. Model performance was assessed using the coefficient of determination ($R^2$, with the mean calculated from five training runs for each configuration. The results are presented in Table 6.

**Table 6:** Hyperparameter sensitivity analysis results on $R^2$.

Learning rate	Adagrad	RMSprop	Adam	SGD	Adamax	NAdam
0.001	0.145	0.956	0.959	0.927	0.844	0.965
0.005	0.614	0.942	0.959	0.878	0.904	0.909
0.01	0.722	0.911	0.948	0.866	0.943	0.900

Table 6 indicates that the model’s performance exhibits significant sensitivity to both the learning rate and the choice of optimizer. Across all tested combinations, the model achieved its highest $R^2$ value of 0.965 when the learning rate was 0.001 and the NAdam optimizer was employed. The Adam optimizer also performed commendably at learning rates of 0.001 and 0.005, with $R^2$ values exceeding 0.959 in both instances. RMSprop and Adamax demonstrated relatively consistent performance across the different learning rates, with $R^2$ values exceeding 0.959 in most cases. In contrast, Adagrad performed significantly worse than the other optimizers at all learning rates, which may be attributed to its gradient accumulation method being unsuitable for the current task. The SGD optimizer’s performance varied considerably with the learning rate, and its overall performance was inferior to that of optimizers based on adaptive gradients.

**Fig. 8:**
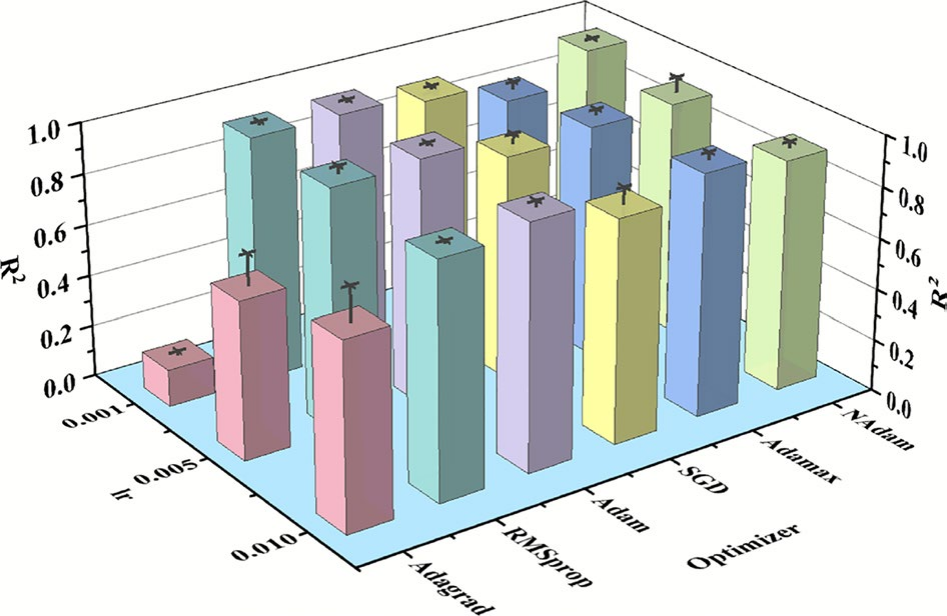
Visualization of $R^2$ values and errors of the SyBN model under different learning rate and optimizer combinations.

To better illustrate the sensitivity of hyperparameters, Fig. 8 presents the model’s $R^2$ values across various learning rates and optimizer combinations, accompanied by error bars.

Figure 8 illustrates the effect of various learning rates and optimizer combinations on the performance (mean $R^2$ value) of the SyBN model using 3D bar charts. The height of each bar represents the average $R^2$ value for a given combination, while the error bar above each bar indicates the range of fluctuation, i.e., the error value, based on five training results for that combination. The length of the error bar reflects the stability of the model’s performance under each hyperparameter configuration: shorter error bars indicate more stable and repeatable performance, while longer error bars suggest greater fluctuation and higher uncertainty in the results. The figure provides the following insights:

Among the tested optimizers, NAdam exhibited superior performance at a learning rate of 0.001, achieving a high coefficient of determination ($R^2$) of 0.965 and a minimal error of 0.005425. This combination indicates that the model configuration is both highly accurate and stable. In contrast, the Adagrad optimizer consistently yielded low $R^2$ values across all tested learning rates. Although it demonstrated some stability at a lower rate of 0.001 with an error of 0.014825, its overall predictive performance remained suboptimal.

The performance of the Stochastic Gradient Descent (SGD) optimizer fluctuated significantly, particularly at learning rates of 0.005 and 0.01, which produced large error bars of 0.03855 and 0.064, respectively, indicating instability. Conversely, the Adam and RMSprop optimizers showed robust performance, exhibiting high $R^2$ values and relatively small error bars across most learning rates. For instance, at learning rates of 0.001 and 0.005, Adam achieved an $R^2$ value of approximately 0.959 with low corresponding errors of 0.011175 and 0.010975, respectivel, reflecting its strong and reliable performance.

From the perspective of the learning rate, better-performing optimizers typically exhibit improved or comparable performance with lower learning rates. In contrast, some optimizers experience a slight reduction in performance when the learning rate is increased to 0.01. This observation suggests that, for training the SyBN model, adopting a smaller learning rate facilitates model convergence and enhances performance.

In conclusion, the NAdam and Adam optimizers demonstrated the best performance during SyBN model training, particularly at lower learning rates. Based on the sensitivity analysis results, NAdam is selected as the default optimizer for SyBN model training in this study, with a learning rate of 0.001. The optimal configuration must balance computational efficiency with model performance, and this analysis provides a critical foundation for the hyperparameter tuning of the SyBN model.

### Interpretability analysis

#### The principle of SHAP

This study introduces the SHAP (SHapley Additive exPlanations) method^47^ to validate the reliability of the SyBN model’s feature importance assessment. SHAP is based on game-theoretic Shapley value theory, which quantifies the marginal contribution of each feature to the model’s prediction.

For sample $\textbf{x}$ and model *f*, the SHAP value $\phi _j(\textbf{x})$ quantifies feature $x_j$’s contribution:

19 $$\begin{aligned} f(\textbf{x}) = \phi _0 + \sum _{j=1}^{p} \phi _j(\textbf{x}) \end{aligned}$$

where $\phi _0$ denotes the baseline value and *p* represents feature dimensionality.

The Shapley value considers feature *j*’s marginal contribution across all possible feature subsets:

20 $$\begin{aligned} \phi _j(\textbf{x}) = \sum _{S \subseteq N \setminus \{j\}} \frac{|S|! (p-|S|-1)!}{p!} [f_{\textbf{x}}(S \cup \{j\}) - f_{\textbf{x}}(S)] \end{aligned}$$

where $N = \{1, 2, \ldots , p\}$ denotes the feature index set and *S* represents feature subsets excluding *j*.

Global feature importance averages absolute SHAP values across all samples:

21 $$\begin{aligned} \text {SHAP}_j^{\text {global}} = \frac{1}{n} \sum _{i=1}^{n} |\phi _j(\textbf{x}_i)| \end{aligned}$$

#### Feature importance analysis

The purpose of this section is to assess the relative importance of each input feature of the SyBN model in predicting the burst pressure of corroded pipelines. Accurate identification of key influences is essential for model validation and to guide engineering practice. The SyBN framework quantifies feature contributions through a Bayesian feature-weighting mechanism integrated into its BFW-NN component. To validate the reasonableness of the Bayesian weight assignment, this study employs the model-independent SHAP interpretation method. SHAP evaluates the average impact of each feature on the overall model output by calculating the Shapley value. Comparing the Bayesian feature weights with the SHAP values allows for cross-validation of the reliability of feature importance determination within the model from different perspectives.

**Fig. 9:**
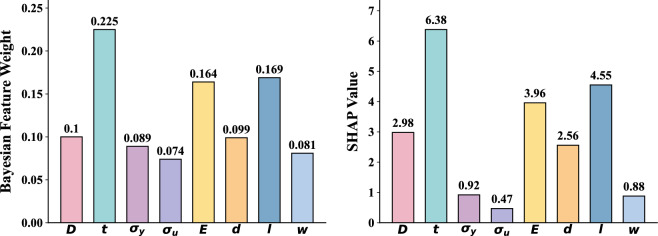
Bayesian feature weights and SHAP values of input features.

Figure 9 presents the Bayesian average feature weights alongside SHAP global importance values. The results indicate that wall thickness (*t*) exhibits the highest importance across both metrics, followed by the modulus of elasticity (*E*) and defect length (*l*). This finding aligns well with the engineering common sense regarding pipeline structural integrity and the impact of defects. In contrast, ultimate tensile strength ($\sigma _u$) and defect width (*w*) demonstrate relatively lower importance. Notably, the Bayesian weights and SHAP values show significant agreement in the relative ordering of feature importance. This mutual corroboration from independent interpretive methods strongly supports the rationality and effectiveness of the Bayesian feature-weighting mechanism within the SyBN model for identifying key predictor variables. By combining Bayesian probabilistic modeling with SHAP a posteriori analysis, this study enhances the credibility of feature importance analysis.

## Conclusion and future work

This study presents a novel SyBN framework for interpretable and accurate prediction of the residual strength of corroded pipelines. By integrating a BFW-NN with a DSR module, and coordinating them through an adaptive gating mechanism, SyBN achieves a balanced fusion of predictive accuracy and model transparency. Experimental validation on a benchmark dataset demonstrates that SyBN significantly outperforms traditional machine learning methods and ensemble models, achieving an R^2^ of 0.966, while providing concise symbolic expressions that reveal underlying relationships between pipeline parameters and failure pressure.

The interpretable nature of SyBN offers substantial benefits for SHM applications, where engineers often require not only accurate estimations but also explicit, physically meaningful models to support risk-informed maintenance, inspection planning, and failure prevention. Specifically, regarding the engineering problem of pipeline integrity assessment, SyBN resolves the conflict between high-precision requirements and the need for transparent decision-making. Its symbolic outputs facilitate rapid onsite verification, while its uncertainty quantification aids in optimizing maintenance schedules under data scarcity. The agreement between Bayesian-derived feature weights and SHAP-based post hoc explanations further strengthens the credibility of the model’s reasoning process, making it a practical tool for field deployment in safety-critical infrastructure systems.

Despite these advancements, this study remains limited by its reliance on static geometric data, which precludes the modeling of time-dependent corrosion growth rates. Additionally, the current dataset does not encompass environmental variables such as soil chemistry and temperature that significantly influence degradation kinetics. Future work will focus on extending the framework to handle real-time data from in-situ monitoring systems, incorporating multi-source sensor data (e.g., acoustic emission, vibration), and expanding symbolic modeling to encompass time-dependent degradation patterns. Furthermore, integration with digital twin platforms may enable adaptive, interpretable diagnostics for pipeline networks operating under variable loading and environmental conditions. The proposed SyBN framework lays a promising foundation for advancing explainable AI methods in the SHM domain.

## Data Availability

The datasets used and/or analysed during the current study are available from the corresponding author on reasonable request and will be upload to GitHub after acceptance.
